# Fear of falling and associated factors among older people living in Bahir Dar City, Amhara, Ethiopia- a cross-sectional study

**DOI:** 10.1186/s12877-021-02534-x

**Published:** 2021-10-21

**Authors:** Gebremeskel Birhanie, Haimanot Melese, Gebrerufael Solomon, Berihu Fissha, Molla Teferi

**Affiliations:** 1 Department of Physiotherapy, School of Medicine, College of Health Sciences, and Tibebe Giyon Specialized Hospital, Bahir Dar University, Bahir Dar, Ethiopia; 2 Department of Physiotherapy, School of Medicine, College of Health Sciences, and Ayder Comprehensive Specialized Hospital, Mekelle University, Mekelle, Ethiopia; 3 Department of Physiotherapy, School of Medicine, College of Health Sciences, and Aksum Referral Hospital, Aksum University, Aksum, Ethiopia; 4 Department of Public Health, School of Public Health, College of Health Sciences, and Ayder Comprehensive Specialized Hospital, Mekelle University, Mekelle, Ethiopia

**Keywords:** Associated factors, Fear of falling, Prevalence, And older people

## Abstract

**Background:**

Fear of falling (FOF) is the most common public health problem, which can lead to loss of confidence, reducing physical and social activities, depression, loss of mobility, increased risk of falls, physical weakness, and strong negative impact on an older people’s quality of life. However, studies in developing country were lacking, particularly in the study area. Therefore, the aim of the current study was to fill this gap in the study area in particular and the country in general. The purpose of the current study was to assess the prevalence and associated factors with fear of falling among older people 60 years and older who were living in Bahir Dar city, Ethiopia.

**Methods:**

A community based cross sectional study design was conducted with a total sample size of 527 participants and multistage random sampling technique was used to select the study participants. The fall efficacy scale tool was used to develop the questionnaire. Data were coded, cleaned and entered into SPSS version 23 for analysis. Multi-collinearity and model fitting were checked. In bivariate logistic regression analyses, variables with *p*-value< 0.25 were considered as potential candidates for multivariable logistic regression analyses. A variable with *p*-value< 0.05 at 95% CI was considered as statistically significant. Finally, the odds ratio and 95% confidence interval were estimated and interpreted.

**Results:**

A total of 481 participants was included in this study. The prevalence of fear of falling among the older people was 59.9% (95% CI; 55.7–64.4). Fear of falling was significantly associated with the following variables:- advanced age (AOR = 4.01, 95% CI; 1.65–9.74), female (AOR = 4.25, 95% CI; 2.25–8.01), lower education level (AOR = 2.77, 95% CI; 1.12–6.82), anxiety [AOR = 9.03, 95% CI; 4.78–17.07), confirmed medical conditions (AOR = 2.01, 95% CI; 1.03–3.91) and walking aids used (AOR = 13.82; 95% CI; 5.21–36.63).

**Conclusions:**

A moderate prevalence of fear of falling was observed. The major associated factors were advanced age, being female, lower educational level, anxiety, confirmed medical conditions and walking aids used. Hence, we recommend the need of rehabilitation programs that enable healthy aging and further rigor research is recommended.

**Supplementary Information:**

The online version contains supplementary material available at 10.1186/s12877-021-02534-x.

## Background

Fear of falling (FOF) is an ongoing concern about falling that ultimately limits the undertaking of daily activities and also known as a post-fall syndrome, or post-fall phobia which is defined as a fearful expectation of a fall [1, 2]. Fear of falling is one of a consequence to fall [3].

It is a major health problem among older people living in the communities who have and have not a history of falling [4–7]. Recent studies showed that 26–55% of older people can experience the FOF [8, 9]. The reported prevalence of FOF varied between 3 and 85% [5]. In the U.S.A, FOF is a common problem among older people, with an incidence ranging from 21 to 85% [10] and approximately one-third of them had activity limitations [11]. In Africa study conducted among older people living in the community of Egypt, prevalence rate of FOF was 64.4% [12].

Fear of falling is known to be associated with old age, female gender, poor vision, frailty, previous history of falls, lower levels of economic resources, poor health, decreased physical function or mobility, the presence of environmental hazards, decreased social contacts, and living alone, depression, anxiety, and a reduced ability perform tasks associated with everyday living [3, 5, 12–17].

Older people with FOF often change their gait, decrease their activity, or attempt to use assistive devices to prevent falling [13]. It may leads to loss of confidence, reducing both physical and social activities/interaction, depression, loss of mobility and independence, risk of future falls and associated mortality, increased physical weakness, and the risk of nursing home admission. These consequences can have a negative impact on older people’s quality of life and economic burden on their families/ or caregivers [16].

Although extensive research was conducted on the burden and determinants in developed countries, there was limited research on FOF among older people living in developing countries, including sub- Saharan Africa countries like Ethiopia. Thus, the present study aimed to assess the prevalence and associated factors of FOF among older people living in the city of Bahir Dar, Ethiopia.

## Methods

### Study area and study period

Bahir Dar is the capital city of Amhara national, regional, state, located in the northwestern part of Ethiopia, at a distance of 565 km far from Addis Ababa. According to the Ethiopian central statistics agency, the total population of the city is ~ 180,174 people in 2007 [18]. Its astronomical location is 11°35′ north latitude, 37°23′ east longitude and 1799 m/5902 ft. above sea level [19]. The administration of the city has six (6) sub city and a total of 26 kebeles. The study was conducted on older people living in the city of Bahir Dar, Ethiopia, from April 1 to May 15, 2019.

### Study design and population

A community-based cross-sectional study design was conducted. All older people aged 60 and older who live in the city of Bahir Dar have been the source of the population. All older people 60 years and older living in selected kebeles during the study period were included. Older people who were not able to communicate and unable to walk independently, with or without a walking support were excluded.

### Sample size determination

For the first objective, based on the data obtained from Bahir Dar city administration bureau of labor and social affairs from a survey conducted in 2018, the total number of older people who were living in the city was 3341 and the profile of all older people living in the city was given by name, age, sex and full address [19]. The sample size was determined by estimating 5% margin of error, 95% confidence interval (alpha = 0.05) and the prevalence of FOF was (64.4%) attributable to a recent similar study in Africa, Egypt 2018 [12]. Based on this assumption, the sample size was determined by using single population proportion formula;

$$\mathrm{n}=\frac{{\mathrm{z}}^2\mathrm{p}\;\left(1\hbox{-} \mathrm{p}\right)}{{\mathrm{d}}^2}$$

Where; n = initial sample size, p = proportion of success, that is the prevalence of FOF in older people, (1-p) = not a proportion of success, that is not FOF in older people, Z a/2 = critical value for normal distribution at 95% CI (1.96) (Z-value at alpha = 0.05), d = margin of error that is acceptable, 5% (0.05).

$${\displaystyle \begin{array}{c}\mathrm{n}=\frac{(1.96)^2\;\left(0.644\times 0.356\right)}{(0.05)^2}\\ {}\mathrm{n}=352.29\approx 353\end{array}}$$

By using population correction formula to get optimum sample size N = [n/ (1 + n/N)] = [353/ (1 + 353/3341)] = 319, since the population is less than 10,000. After Appling 1.5 multiplying factor for the design effect the sample size was 479. By adding 10% non-response rate and minor adjustment the total sample size was 527 older people.

The sample size for objective two was calculated based on the assumption; FOF developed for those who had an exposed with depression is being 53.6% and those who had no depression is being 30.6% FOF [6]. Assuming 5% margin of error (d), 95% confidence level (alpha, α = 0.05, two tailed) and 80% power to detect the assumed difference. This was calculated using the Epi Info 7 statistical software to get the optimum sample size with ratio = 0.22, the final calculated sample size was 271 on Fleiss w/CC. After adding 10% non-response rate and minor adjustment the total sample size was 298 older people. Therefore, the total sample size of this study was 527 older people.

### Sampling technique and procedure

**Fig. 1:**
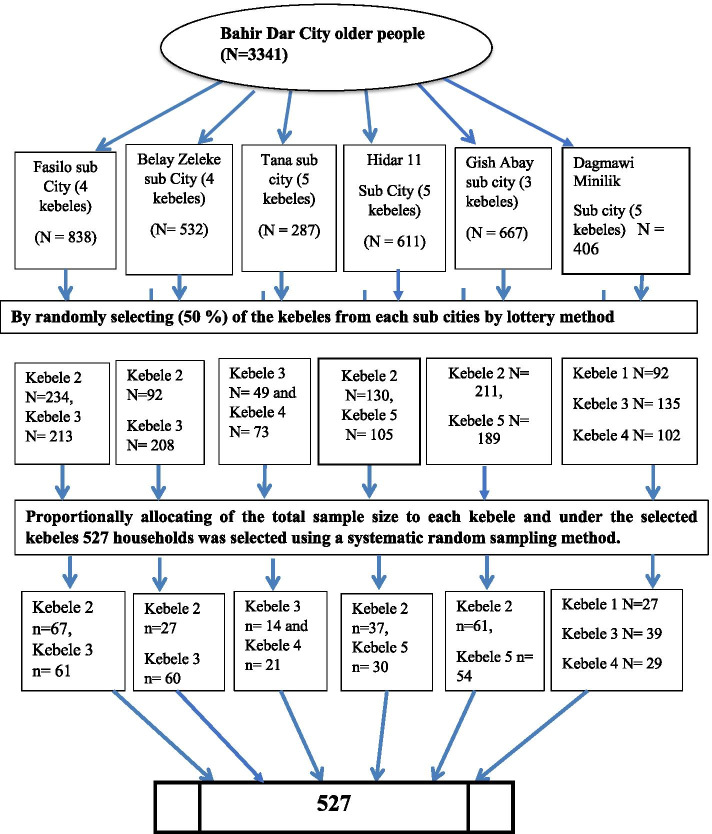
Schematic presentation of sampling procedure among older people in Bahir Dar City, Ethiopia

A preliminary survey was conducted in 2018 by the Bahir Dar city labor and social affairs administration office to find out about the total number of older people residing in Bahir Dar city and the total amount of older people was found to be 3341. The administration of the city has six (6) sub city (kefleketema) and a total of 26 Kebeles. A multistage random sampling technique was employed to select the study participants. In the first stage, 13 out of 26 kebeles (50% of the total area) were selected by simple random sampling technique (Fig. 1). A total number of households were obtained from the respective administrative areas and used to calculate the sampling fraction. Sampling interval, K^th^ was calculated by N/n, 1833/527 = 3.47 **≈** 4. The starting household was identified by the lottery method and every 4th household elderly individual from each kebele was recruited for the study until the appropriate sample size was reached. Occasionally, when two or more individuals were eligible in a household, only one was selected by lottery method.

### Data collection tool, methods and procedures

The data were collected by interviewing the participants using a semi-structured questionnaire that was adapted from related literatures (Additional file 1) and standard questionnaire was adopted from tools (i.e. FES-I scale, GDS-SF scale, GAD-7 scale and Katz index of independent in ADL tool) [19–24]. Falls Efficacy Scale-International (FES-I):- contains 16 items, with scoring based on a 4-point. Total score ranges from 16 to 64 points; 16 meant no fear, 17–32 little fear, 33–48 moderate fear, and 49–64 intense fear [25, 26]. The Geriatric Depression Scale–Short Form (GDS-SF) was used to screen for depressive symptoms in this study. The 15 items in the GDS-SF were extracted from the original 30-item GDS. Interpretation of the score (a score of 0 to 5 is normal, a score > 5 suggests depression and a score ≥ 10 is almost always indicative of depression) [22]. The original General Anxiety Disorder (GAD-7) was applicable in measuring the respondents’ level of anxiety and consists of seven multiple choice questions. Total score ranges from 0 to 4 is minimal anxiety, 5–9 mild anxiety, 10–14 moderate anxiety and a score greater than 15 has severe anxiety [23]. The adopted questionnaire was included, sociodemographic variables (Age, sex, marital status, household income, educational level and body mass index), psychosocial factors (anxiety, depression, social support and physical exercise), fall-related factors (history of falling, and Injury with fall), disease and treatment related factors (disease conditions, Activity of daily life (ADL), number of co-morbidity and number of type of medications used) and environmental level factors (discomfort in neighborhood environment and walking aids) (Additional file). Data collectors were trained and the informed consent was obtained from each participant. The weight scale tool specification TIANSHA = 2003A, capacity: 11 lb. / 5 kg - 400 lb. / 180 kg / 28 St 8 lbs., Battery: CR2032 Lithium Battery was used to measure weight and height were measured using tape measures tool specification WIN TAPE, (FT-070). Height and weight were measured after the end of the interview. Data collectors and supervisors were trained.

### Operational definitions

#### Fear of falling

Is positive when a cut-point for FOF as scores of FES-I was > 23. This means a total score of FES-I scale is from 24 to 64 on the numerical rating scale [18, 20, 21].

#### Older people

The age at which a person becomes 60 years and above refer to the older people [4, 6, 12, 27–32].

#### Physical exercise

Is any kind of moderate – intensity exercise (such as walking, cycling, sports or planned exercise and strength exercise) done at least 150 min per week [33, 34].

### Data quality control issues

To ensure the quality of data all the necessary measures were done before, during and after the actual data collection. A valid and reliable instrument was used for the data collection. All data collectors and supervisors were given one-day training about the purpose of the study, details of the data collection instrument (questionnaire), interviewing techniques, the importance of privacy and insuring confidentiality of the respondents.

The original English version of the questionnaire was translated into Amharic and re-translated back into English to maintain its consistency. Pretest was conducted on 5% of the total sample size. The questionnaire was reviewed and checked for completeness, accuracy and consistency by supervisors and investigators.

### Data processing and analysis

The collected data were coded and cleaned and then it was entered into SPSS version 23 packages for analysis. Descriptive statistics, frequency and percentages, mean and standard deviations were used to describe the findings. Multi-collinearity was checked by variance inflation factor (VIF) cutoff point < 10. Model fitness was checked by Hosmer- Lemeshow test. Bivariate logistic regression analysis was used to determine which variables had an association with FOF. In bivariate logistic regression analyses, variables with *p*-value< 0.25 were considered as potential candidates for multivariable logistic regression analyses. All possible predictors which were significant in the bivariate analysis were included in the multiple logistic models. A variable with *p*-value< 0.05 at 95% CI was considered as statistically significant. Finally, AOR with 95% CI at *p* - value of < 0.05 was reported.

## Results

### Socio-demographic and personal characteristics among older people 60 years and older

Among the total 527 study participants, 481 of them completed the interview with a 91.2% response rate. The majority of the participants were female (*n* = 251, 52.2%) with a mean age of 67.9 years (SD = 7.6). Almost half (*n* = 271, 56.3%) of were married with the median monthly household income of 890 Ethiopian Birr. Four hundred nineteen participants (87.1%) declared that they completed lower level of education and most participants had normal BMI score (*n* = 398, 82.7%) (Table 1).

**Table 1:** The descriptive characteristics of FOF among study participants in Bahir Dar city, Ethiopia (*n* = 481)

Variables	Categories	Frequency, n (%)	FOF	
			Yes, n (%)	No, n (%)
Sex	Female	251 (52.2)	177 (70.5)	74 (29.5)
	Male	230 (47.8)	111 (48.3)	119 (51.7)
Age	60–70	351 (73.0)	170 (48.4)	181 (51.6)
	≥71	130 (27.0)	118 (90.8)	12 (9.2)
Marital status	Currently Married	271 (56.3)	141 (52.0)	130 (48.0)
	Currently Unmarried	210 (43.7)	147 (70.0)	63 (30.0)
Body mass index (BMI)	< 18.5	11 (2.3)	8 (72.7)	3 (27.3)
	18.5–24.9	398 (82.7)	229 (57.5)	169 (42.5)
	≥25	72 (15.0)	51 (70.8)	21 (29.2)
Monthly Income	≤ETB1752 (US$ 60.98)	304 (63.2)	195 (64.1)	109 (35.9)
	≤ETB1752 (US$ 60.98)	177 (36.8)	93 (52.5)	84 (47.5)
Education status	Lower level education	419 (87.1)	270 (64.4)	149 (35.6)
	Higher level education	62 (12.9)	18 (29.0)	44 (71.0)
Anxiety	Yes	224 (46.6)	190 (84.8)	34 (15.2)
	No	257 (53.4)	98 (38.1)	159 (61.9)
Depression	Yes	419 (87.1)	272 (64.9	147 (35.1)
	No	62 (12.9)	16 (25.8)	46 (74.2)
Social support	Yes	330 (68.6)	201 (60.9)	129 (39.1)
	No	151 (31.4)	87 (57.6)	64 (42.4)
Physical exercise	Yes	80 (16.6)	24 (30.0)	56 (70.0)
	No	401 (83.4)	264 (65.8)	137 (34.2)
History of falling	Yes	103 (21.4)	90 (87.4)	13 (12.6)
	No	378 (78.6)	198 (52.4)	180 (47.6)
Injury with fall	No	52 (10.8)	43 (82.7)	9 (17.3)
	Yes	51 (10.6)	47 (92.2)	4 (7.8)
Activity of daily life	Functional limitation	4 (0.8)	4 (100.0)	0 (0.0)
	No functional limitation	477 (99.2)	284 (59.5)	193 (40.5)
Number of types of medications	0–3	439 (91.3)	250 (56.9)	189 (43.1)
	≥4	42 (8.7)	38 (90.5)	4 (9.5)
Confirmed medical problems	Yes	276 (57.4)	209 (75.7)	67 (24.3)
	No	205 (42.6)	79 (38.5)	126 (61.5)
Number of medical problems	1	141 (29.3)	92 (65.2)	49 (34.8)
	≥2	135 (28.1)	117 (86.7)	18 (13.3)
Hypertension	Yes	144 (29.9)	109 (75.7)	35 (24.3)
	No	337 (70.1)	179 (53.1)	158 (46.9)
HIV/AIDS	Yes	9 (1.9)	5 (55.6)	4 (44.4
	No	472 (98.1)	283 (60.0)	189 (40.0)
Stroke	Yes	30 (6.2)	30 (100.0)	0 (0.0)
	No	451 (93.8)	258 (57.2)	193 (42.8)
Diabetes Mellites	Yes	94 (19.5)	73 (77.7)	21 (22.3)
	No	387 (80.5)	215 (55.6)	172 (44.4)
Back Pain	Yes	24 (5.0)	16 (66.7)	8 (33.3
	No	457 (95.0)	272 (59.5)	185 (40.5)
Pneumonia	Yes	3 (0.6)	3 (100)	0 (0.0)
	No	478 (99.4)	285 (59.6)	193 (40.4)
Osteoporosis	Yes	31 (6.4)	20 (64.5)	11 (35.5)
	No	450 (93.6)	268 (59.6)	182 (40.4)
Arthritis	Yes	44 (9.1)	35 (79.5)	9 (20.5)
	No	437 (90.9)	253 (57.9)	184 (42.1)
Eye problem	Yes	35 (7.3)	31 (88.6)	4 (11.4)
	No	446 (92.7)	257 (57.6)	189 (42.4)
Heart problem	Yes	20 (4.2)	19 (95.0)	1 (5.0)
	No	461 (95.8)	269 (58.4)	192 (41.6)
Kidney problem	Yes	22 (4.6)	19 (86.4)	3 (13.6
	No	459 (95.4)	269 (58.6)	190 (41.4)
Asthma	Yes	22 (4.6)	15 (68.2)	7 (31.8)
	No	459 (95.4)	273 (59.5)	186 (40.5)
Gastritis	Yes	10 (2.1)	7 (70)	3 (30)
	No	471 (97.9)	281 (59.7)	190 (40.3)
Environment comfort	Yes	345 (71.7)	208 (60.3)	137 (39.7)
	No	136 (28.3)	80 (58.8)	56 (41.2)
Walking aid used	Yes	141 (29.3)	134 (95.0)	7 (5.0)
	No	340 (70.7)	154 (45.3)	186 (54.7)

### Psychosocial level characteristics

Of the total study participants, (*n* = 224, 46.6%) had anxiety and about (*n* = 419, 87.1%) had depression. Four hundred one, 83.4% participants reported that they were not doing regular physical exercise. Most of the participants had social support (*n* = 330, 68.6%) (Table 1).

### Fall-related factors characteristics

Of the total study participants, (*n* = 103, 21.4%) of the participants had a history of experienced falling. Fifty one (10.6%) of the respondents was hurt by with fall (Table 1).

### Disease or treatment related characteristics

Almost all, (*n* = 477, 99.2%) of the study participants indicated that there was no functional limitation due to FOF. About (*n* = 276, 57.4%) of the study participants reported that having confirmed medical conditions. One hundred thirty five, 28.1%) of the study participants reported that more than or equal to two medical problems. The commonly reported type of medical problems was hypertension (*n* = 144, 29.9%), diabetes mellitus (*n* = 94, 19.5%) and arthritis (*n* = 44, 9.1%) (Table 1).

### Environmental level characteristics

Among the study participants, (*n* = 345, 71.7%) were declared that they were living in a comfortable environment and about (*n* = 141, 29.3%) participants were using walking aids (Table 1).

### Prevalence of fear of falling among older people 60 years and older in Bahir Dar City

The overall prevalence of FOF among older people 60 years and older who were living in Bahir Dar city was (*n* = 288, 59.9, 95% CI, 55.7–64.4%). Among those who develop FOF (*n* = 177, 70.5%) were females. The prevalence of FOF (*n* = 118, 90.8%) was ≥71 year of age. Those who had a history of falling (*n* = 90, 87.4%) also complained about FOF in the past 12 months.

The prevalence of FOF among study participants with lower levels of education was (*n* = 270 64.4%). Of the total study respondents, the prevalence of FOF with had confirmed medical problems was (*n* = 209, 75.7%). The prevalence of FOF among those with had hypertension was (*n* = 109, 75.7%), DM (*n* = 73, 77.7%), and eye problems (*n* = 31, 88.6%). Those who had anxiety complained about (*n* = 190, 84.8%) of FOF in the last 2 weeks.

### Severity of level of FOF among older people

**Fig. 2:**
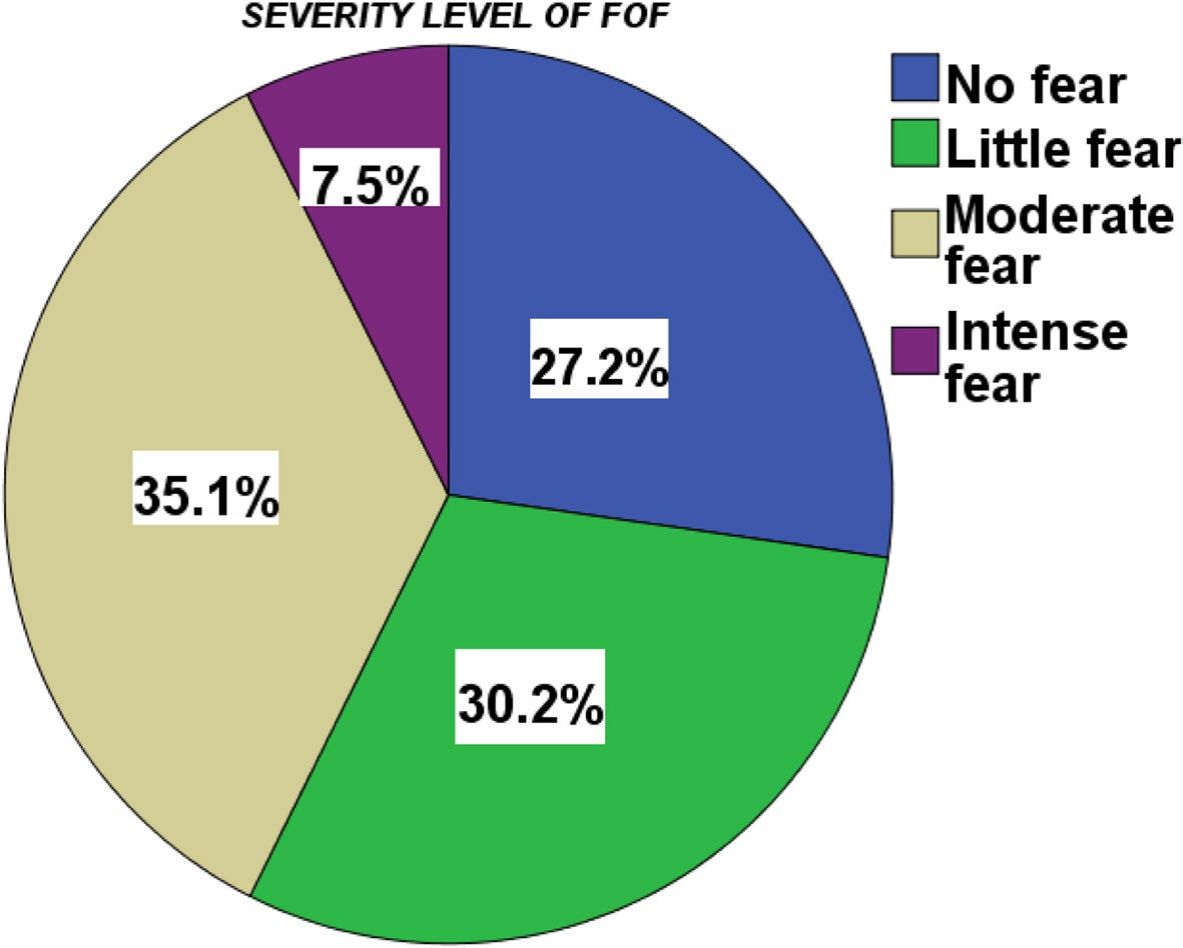
severity of FOF among older people in Bahir Dar city, Ethiopia

Of the total participants, (*n* = 169, 35.1%) of older people were moderately developed FOF. (Fig. 2).

### Factors associated with fear of falling

Variables that showed a substantial association with FOF in the bivariate analysis were age, sex, educational status, marital status, household income, anxiety, depression, physical exercise, history of falling, injury with fall, confirmed medical conditions, number of comorbidities, the number types of medications used, and walking aids used.

The variables that showed statistically significant association with FOF during the multivariate analysis were advanced age, female, lower educational level, anxiety, confirmed medical conditions and walking aids used with *p*-value < 0.05.

The result of this study showed that, being age ≥ 71 years’ of age were approximately four times more likely higher than age in between 60 and 70 years’ old (AOR = 4.0; 95% CI, 1.65–9.74). It was found that female respondents were four times more likely to develop FOF than male respondents (AOR = 4.2; 95% CI, 2.25–8.01).

Older people who completed the lower level education were almost three times more likely to experience FOF than those who completed higher levels of education (AOR = 2.7; 95% CI, 1.12–6.82).

The result of the current study also showed that elderly people with medical problems were twice as likely to be affected by FOF as those without medical problems (AOR = 2.0; 95% CI, 1.03–3.91). Anxiety was nine times higher FOF than don’t have anxiety (AOR = 9.0; 95% CI, 4.784–17.07). Older people who used walking aids was nearly 14 times more likely to be affected by FOF than older people who don’t have use of it (AOR = 13.8; 95% CI, 5.21–36.63) (Table 2).

**Table 2:** Factors associated with FOF among older people 60 years and older living in Bahir Dar city, Ethiopia (*n* = 481)

Variables		Bivariate		Multivariate	
		COR (95% CI)	***P***-value	AOR (95% CI)	***P***-value
Sex	Male	1.000		1.000	
	Female	2.5 (1.76–3.73) *	0.000	4.2 (2.25–8.01) **	< 0.001
Age	60–70	1.000		1.000	
	≥71	10.4 (5.57–19.65) *	0.000	4.0 (1.65–9.74) **	0.002
Education status	Lower level education				
	Higher level education				
Marital status	Currently Married	1.000		1.000	
	Currently Unmarried	2.15 (1.47–3.14) *	0.000	1.2(0.71–2.30)	0.399
Monthly Income	> ETB 1752 (US$ 60.98)	1.000		1.000	
	≤ ETB1752 (US$ 60.98)	1.6 (1.10–2.35) *	0.013	0.5(0.31–1.09)	0.091
Anxiety	No	1.000			
	Yes	9.0 (5.82–14.12) *	0.000	9.0(4.78–17.07)**	< 0.001
Depression	No	1.000		1.000	
	Yes	5.3 (2.91–9.72) *	0.000	1.5 (0.72–3.35)	0.256
Physical exercise	Yes	1.000		1.000	
	No	4.4 (2.67–7.56) *	0.000	1.4 (0.69–3.18)	0.308
History of fall	No	1.000		1.000	
	Yes	6.2 (3.40–11.64) *	0.000	2.1 (0.63–7.23)	0.223
Injury with fall	No	1.000			
	Yes	2.4 (0.70–8.56) *	0.158	0.2 (0.04–1.06)	0.059
Confirmed medical condition	No	1.000		1.000	
	Yes	4.9 (3.35–7.37) *	0.000	2.0 (1.03–3.91) **	0.038
No of comorbidities	1	1.000		1.000	
	≥2	3.4 (1.89–6.34)	0.000	0.5 (0.23–1.25)	0.155
No of type of medication used	0–3				
	≥4	7.1 (2.52–20.47) *	0.003	2.0 (0.45–8.89)	0.362
Walking aids	No				
	Yes	23.1 (10.50–50.91)*	0.000	13.8(5.21–36.63) **	< 0.001

NB: *COR* odds ratio, *AOR* adjusted odds ratio, * = significant association (on bivariate), ** = significant association (on multivariate, *p*-value< 0.05), 1.000 = Reference. Model of fitness test (Chi-square = 12.381, df = 8, sig = 0.135)

## Discussion

This study was conducted to determine the prevalence and associated factors of fear of falling among older people 60 years and older who were living in Bahir Dar City. The overall prevalence of fear of falling among older people aged 60 years and older living in Bahir Dar city was found to be (*n* = 288, 59.9, 95% CI, 55.7–64.4%). This result indicates that FOF is a common concern for older people living in Bahir Dar. The finding of this study will give a great emphasis, which helps to address realted health problems as aconsquence of FOF in older population at large. Besides, it provides scientific data so as to create a wareness on prevention or health promotion, to reduce the occurrence of fall, to minimize costs and secondary complications. The current study showed FOF severity classification, 7.5% reported no FOF, 31.1% reported that little fear, 35.1% reported moderate fear and 27.3% reported intense FOF.

The prevalence of the current study was comparable to the similar study conducted in the Longtan community in Beijing, China and Mansoura city, Egypt, which was 58.8 and 64.4% respectively [12, 35]. This similarity in these studies could be due to the use of similar cross-sectional study design and the use of the FES-I assessment tool, and the data were collected through interviews with participants.

However, the prevalence of this study was lower than that of Diamantina, Brazil (90.4%), Rio de Janeiro, Brazil (95.2%), Sao Paulo, Brazil (66.5%), Iran (90.3%) and Korea (70.6%) [4, 28, 29, 36, 37]. This variance may be due to discrepancies in methodology, sample size, living environment, and tools used between these studies and our study, which may be one potential explanation for this discrepancy. For instance, a study in Rio de Janeiro, Brazil, was conducted among 742 participants using the local Brazilian version of the falls efficacy scale international outcome assessment method to assess FOF. Similarly, a study in Sao Paulo, Brazil, of 170 older people using the Brazilian version of the falls efficacy scale-international outcome measurement tool for FOF assessment and another study in Korea, of 4164 older people using the longitudinal follow-up study design.

The prevalence of this study was higher than the cross sectional study conducted in the UK (19%), Spain (41.5%), India (33.2%), Taiwan (53.4%) and Nigeria (34.4%) [1, 6, 38–40]. This disparity may be due to the different sample size, the outcome assessment instrument, the methodology, as well as the educational and economic differences.

For instance, a study conducted in Spain among 640 participants using a longitudinal prospective study design and also, instead of using FES-I outcome measurement method, FOF was investigated using a dichotomous yes / no question: “Are you afraid of falling?” Similarly, a study in Taiwan using a community based survey of 3824 participants and FOF was investigated using the same dichotomous yes / no question.

The findings of this study have shown that FOF has a significant association with advanced age, gender, lower educational level, anxiety, confirmed medical conditions and walking aids used.

A substantial association between FOF and gender was found in this study, with females 4.2 times more likely to develop FOF than males. This was consistent with a cross-section analysis conducted in the Netherlands, which confirmed that being female was significantly correlated (three times) with the development of FOF [41]. Likewise, other studies done in Hong Kong, China and Korea evidenced that older people have reported a greater FOF in women compared to men [7, 38]. On the other hand, a systematic review study done by Scheffer et al., 2008 also revealed that, being females had more exposed to FOF than male [5]. In addition, the result of this study supported by studies done in Mexico, Spain, Egypt, Netherland, Thailand and UK, the rate of FOF was higher in female subjects than male [12, 31, 32, 38, 39, 42]. This similarity might be due to older women is affected by gender-related factors such as postmenopausal low bone density, faster loss of muscle mass due to reduced hormones [43] and a higher prevalence of chronic non-communicable diseases, and musculoskeletal frailty [44]. On the other side, a studies done in India and Iran, revealed that, no significant relationship was observed between gender and FOF [4, 6]. This might be because of a small sample of female with FOF observed in both studies done in India and Iran and this might make low statistical power for significance of female in both studies.

The findings of the current study showed an important correlation between FOF and age. Older people who are over 70 years of age experience FOF 4.0 times more than younger elderly people. This finding is confirmed by the previous study in Iran, where older people over 70 years of age are 3.2 more likely to have FOF than those under 70 years of age [4]. Similarly, other studies conducted in Taiwan, Egypt, India, Brazil, Japan, Korea, Mexico, and the Netherlands have found that advanced age is a significant predictor of FOF, which is consistent with the findings of this study [1, 6, 12, 14, 29–31, 45]. The outcome of this study can be justified as advanced age is accompanied by loss of physical, psychological, and physiological change in function is accelerated with aging, which can put older people at risk of falling and FOF.

To the contrary, a study done in the UK, Australia, and the USA, indicated that advanced age not significantly associated with FOF [13, 19, 38]. This may be due to the awareness of older people living in the study area about the use of fall and the FOF prevention program, which in turn leads them both mentally and physically strong.

In this study, FOF was associated with anxiety; older people who had anxiety were found to be 9.0 times more likely to experience FOF than those who did not. This finding is consistent with other studies conducted in China, Netherland, and a systematic review study conducted by Abyad et al. in 2017 revealed a significant relationship between anxiety and FOF [7, 14, 46]. This might lead older people to develop feelings of insecurity either to walk or to do an activity and a reason for a reduction of social participation and this loss of personal contact which in turn increase isolation.

The finding of current study showed that FOF has a significant association with education. Lower education was found to be 2.7 times more likely to acquire FOF than those who have completed higher education level. This result is consistent with another study conducted in the UK, which found that older people with lower levels of education reported higher FOF than higher levels of education [38]. It is also supported by studies conducted in Thailand, Iran, India, and two different studies done in Korea and in which older people with lower educational levels had a significant association with FOF [4, 6, 32, 45, 47]. Because of the lower level of education, it may be difficult to establish a coping strategy to minimize FOF, which can lead them to become less active and choose to remain inactive, leading to falling and physical instability.

A significant relation was noted between the presences of confirmed medical health conditions in this current study. Confirmed medical health conditions were found to be 2.0 times more likely to develop FOF than who don’t have. This finding is supported by other studies conducted in Thailand, Taiwan, Netherland, India, Egypt, two different studies in Korea and a systematic review study done by Abyad et al., 2017 revealed that a significant association between older people who have medical problems and FOF [1, 6, 12, 14, 32, 45–47]. This poor health status might lead to develop fatigue, weakness, and loss of self-confidence/self-esteem. This may also lead to loss of motivation associated with a reduction of their activity and social interaction and finally this may be a risk factor for activity limitations due to FOF.

Older people who used walking aids during gait were 13.8 times more likely to be affected with FOF than who had not used walking aids in this current study. This result is supported by another study conducted in Egypt, USA, and UK had noted that FOF showed a significant association with use walking aids among older people living in the community [12, 19, 38]. This might be because of older people with FOF had lost confidence in overcoming challenging situation and obstacles in their environment and this may lead them to reduce to maintain balance or control of gait at work or to perform their tasks and because of this older people may enforce to use walking aids for gait.

### Limitaion of the study

Most of the studies were done in developed countries and it was difficult to compare the result of this study to other studies done in a similar setting.

## Conclusion

The prevalence of fear of falling among older people living in Bahir Dar City is found to be moderate. The findings of the present study showed that fear of falling in the elderly has a major associated with advanced age, female, lower educational level, anxiety, confirmed medical health problems and the use of walking aids. Health office (Ministry of Health), health care providers need to plan and design appropriate for fall and FOF preventive strategies, guidelines or policies as well as allocate appropriate resources for preventive interventions. Health practitioners need to work by providing relevant information to concerned bodies and older people to raise awareness, focus on prevention or health promotion, reduce the risk of falling FOF, minimize costs and associated secondary complications. Further studies with follow up study design recommended.

## Supplementary Information

**Additional file 1.** English version of questionnaire.

## Data Availability

Data will be available upon request from the corresponding author.

## Abbreviations

ADL: Activity of daily life
AOR: Adjusted Odds Ratio
BMI: Body Mass Index
CI: Confidence Interval
COR: Crude Odds Ratio
ETB: Ethiopian Birr
FES-I: Falls Efficacy Scale-International
FOF: Fear of falling
GAD-7: General Anxiety Disorder Seven
GDS-SF: Geriatric Depression Scale–Short Form
HOF: History of fall
ORs: Odds Ratios
SD: Standard Deviation
SPSS: Statistical Package for social sciences
UK: United kingdom
U.S.A: United States of America
WHO: World health organization.
