# Supplementary material for: Fear of falling and associated factors among older people living in Bahir Dar City, Amhara, Ethiopia- a cross-sectional study
Source: BMC Geriatr. 2021 Oct 21;21:586. doi: 10.1186/s12877-021-02534-x (PMC8532299; doi:10.1186/s12877-021-02534-x)
Supplement: Supplementary file 1 — Additional file 1. English version of questionnaire. [file 12877_2021_2534_MOESM1_ESM.docx]

**Additional file 1**

**English version of questionnaire**

Read the following question and fill or circle the space that provide your answer

**Part one: Socio demographic characteristics**

| No | Question | Possible response | Skip |
| --- | --- | --- | --- |
| FD1 | Sex | 1.Male 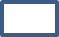 2. Female 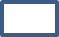 |  |
| FD2 | Age | ________________ In year |  |
| FD3 | What is your marital status? | 1. Married 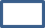 2. Single 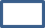  3. Divorce 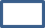 4. widowed 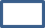  5. Separated 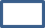 6. Cohabitated 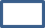 |  |
| FD4 | Weight | _________________________________ |  |
| FD5 | Height | __________________________________ |  |
| FD6 | BMI | Underweight (<18.50)  Normal (18.50-24.99)  Overweight (25 -29.9)  Obese (≥ 30) |  |
| FD7 | What is your highest education level? | Can’t read and write 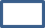 Read and write with out formal education 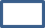 Primary school 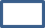 High school 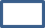 College 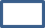 Bachelor’s degree 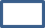 Master degree 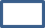 PHD 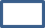 |  |
| FD8 | How much household income do you earn per month | __________________________ETB |  |

**Part two: General Health status**

| FH1 | Do you have any confirmed medical problem? | 1.yes 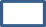 2.No 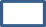 | FH3 |
| --- | --- | --- | --- |
| FH2 | If you answer yes for FH1 specify | _________________________ |  |
| FH3 | Do you take any medicines now? | 1.yes 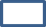 2.No 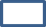 | A1 |
| FH4 | If your answer yes for FH3 specify the number of types of medication per day? | _____________________________ | Skip |
| **Information on Activity of daily life using Katz Index of Independence in Activities of Daily Living** | | | |
|  | **Activities** | | |
| A1 | Do you ask supervision, direction or personal assistance when you bathing? | 1. Yes 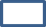 2.No 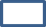 |  |
| A2 | Do you ask supervision, direction or personal assistance when you dressing? | 1. Yes 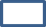 2.No 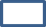 |  |
| A3 | Do you ask supervision, direction or personal assistance when your toileting? | 1. Yes 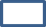 2.No 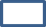 |  |
| A4 | Do you asking supervision, direction or personal assistance when you move and out of bed or moving from bed to chair? | 1. Yes 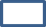 2.No 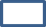 |  |
| A5 | Can you control when your urine or bowel is coming till to reach to toilet? | 1. Yes 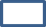 2.No 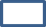 |  |
| A6 | Do you move food from plate into mouth without help? | 1. Yes 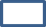 2.No 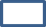 |  |

**Part three: fall-related factors** **associated with fear of falling**

| FF1 | Have you ever experienced a fall within a year ? | 1.Yes 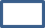 2. No 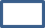 | Skip EF1 |
| --- | --- | --- | --- |
| FF2 | If yes, in question in FF1 how many times experienced? |  |  |
| FF3 | If yes, in question FF1 are you got injury with fall? | 1.Yes 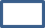 2. No 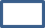 |  |

**Part four: Psychosocial level factor associated with fear of falling**

| EF1 | Did you have the habit of doing physical exercising? | 1.Yes 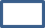 2. No 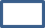 | | | | Skip EF3 |
| --- | --- | --- | --- | --- | --- | --- |
| EF2 | If say yes in question above, how often do you do exercise per week | --------minutes/days------days/week | | | |  |
| EF3 | Do you have any social support from the family/ neighborhood? | 1.Yes 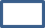 2. No 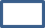 | | | |  |
|  | **Information on depression using Geriatric Depression Scale Short Form (GDS-SF)**  **(please put/circle the answer on the space provided)** | | | | | |
| **Choose the best answer for how you have felt over the past week:** | | | | | | |
| D1 | Are you basically satisfied with your life? | 1.Yes 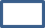 2.No 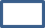 | | | | Skip |
| D2 | Have you dropped many of your activities and interests? | 1.Yes 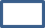 2.No 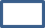 | | | |  |
| D3 | Do you feel that your life is empty? | 1.Yes 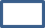 2.No 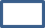 | | | |  |
| D4 | Do you often get bored? | 1.Yes 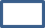 2.No 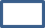 | | | |  |
| D5 | Are you in good spirits most of the time? | 1.Yes 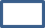 2.No 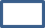 | | | |  |
| D6 | Are you afraid that something bad is going to happen to you? | 1.Yes 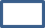 2.No 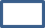 | | | |  |
| D7 | Do you feel happy most of the time? | 1.Yes 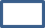 2.No 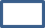 | | | |  |
| D8 | Do you often feel helpless? | 1.Yes 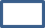 2.No 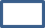 | | | |  |
| D9 | Do you prefer to stay at home, rather than going out and doing new things? | 1.Yes 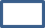 2.No 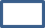 | | | |  |
| D10 | Do you feel you have more problems with memory than most? | 1.Yes 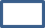 2.No 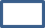 | | | |  |
| D11 | Do you think it is wonderful to be alive now? | 1.Yes 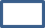 2.No 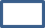 | | | |  |
| D12 | Do you feel pretty worthless the way you are now? | 1.Yes 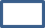 2.No 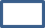 | | | |  |
| D13 | Do you feel full of energy? | 1.Yes 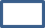 2.No 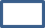 | | | |  |
| D14 | Do you feel that your situation is hopeless? | 1.Yes 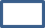 2.No 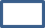 | | | |  |
| D15 | Do you think that most people are better off than you are? | 1.Yes 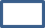 2.No 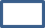 | | | |  |
| **Information on anxiety using Generalized Anxiety Disorder 7-item (GAD-7)**  **(please put/circle the answer on the space provided)** | | | | | | |
| **Over the last 2 weeks, how often have you been bothered by the following problems?** | | Not at all | Several days | More than half the days | Nearly every day |  |
| 1A | Feeling nervous, anxious or on edge | 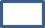 | 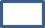 | 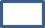 | 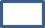 | Skip |
| 2A | Not being able to stop or control worrying | 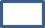 | 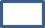 | 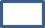 | 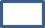 |  |
| 3A | Worrying too much about different things | 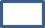 | 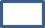 | 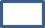 | 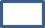 |  |
| 4.A | Trouble relaxing | 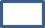 | 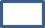 | 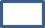 | 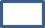 |  |
| 5A | Being so restless that it is hard to sit still | 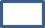 | 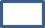 | 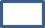 | 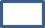 |  |
| 6A | Becoming easily annoyed or irritable | 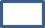 | 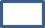 | 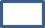 | 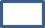 |  |
| 7A | Feeling afraid as if something awful might happen | 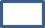 | 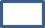 | 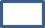 | 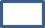 |  |

**Part five: Environmental factors associated with fear of falling**

| E1 | Is it a comfortable space in your home entrance/ hallway, stairs, bathroom, living room, kitchen room to operate and move? | 1.Yes 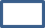 2.No 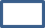 | Skip |
| --- | --- | --- | --- |
| E2 | Is your location a comfortable place to move and to work? | 1.Yes 2.No |  |
| E3 | Do you feel comfortable when you travel to facilities or service centers? (e.g. markets, banks, public transportation, medical facilities, social welfare facilities (including leisure/cultural facilities) | 1.Yes 2.No |  |
| E4 | Are you use walking aids? | 1.yes 2.no | FE1 |
| E5 | If you answer yes for FH4 specify the type of walking aid? | _________________ |  |

**Part six: Information on fear of falling using Falls Efficacy Scale-International (FES-I) scale questions about how concerned you are about the possibility of falling if you did these activity (please put/circle the answer on the space provided)**

|  |  | Not at all concerned | Somewhat concerned | Fairly concerned | Very concerned |  |
| --- | --- | --- | --- | --- | --- | --- |
| FE1 | Cleaning the house (e.g. sweep, vacuum or dust) |  |  |  |  | Skip |
| FE2 | Getting dressed or undressed |  |  |  |  |  |
| FE3 | Preparing simple meals |  |  |  |  |  |
| FE4 | Taking a bath or shower |  |  |  |  |  |
| FE5 | Going to the shop |  |  |  |  |  |
| FE6 | Getting in or out of a chair |  |  |  |  |  |
| FE7 | Going up or down stairs |  |  |  |  |  |
| FE8 | Walking around in the neighbourhood |  |  |  |  |  |
| FE9 | Reaching for something above your head or on the ground |  |  |  |  |  |
| FE10 | Going to answer the telephone before it stops ringing |  |  |  |  |  |
| FE11 | Walking on a slippery surface (e.g. wet or icy) |  |  |  |  |  |
| FE12 | Visiting a friend or relative |  |  |  |  |  |
| FE13 | Walking in a place with crowds |  |  |  |  |  |
| FE14 | Walking on an uneven surface (e.g. rocky ground, poorly maintained pavement) |  |  |  |  |  |
| FE15 | Walking up or down a slope |  |  |  |  |  |
| FE16 | Going out to a social event (e.g. religious service, family gathering or club meeting) |  |  |  |  |  |
|  | Final score |  | | | | |
